# Molecular Indicators of Blood-Brain Barrier Breakdown and Neuronal Injury in Pregnancy Complicated by Fetal Growth Restriction

**DOI:** 10.3390/ijms232213798

**Published:** 2022-11-09

**Authors:** Natalia Misan, Sławomir Michalak, Piotr Rzymski, Barbara Poniedziałek, Katarzyna Kapska, Krystyna Osztynowicz, Mariola Ropacka-Lesiak

**Affiliations:** 1 Department of Perinatology and Gynecology, Poznan University of Medical Sciences, 60-535 Poznan, Poland; 2 Department of Neurochemistry and Neuropathology, Poznan University of Medical Sciences, 60-355 Poznan, Poland; 3 Department of Environmental Medicine, Poznan University of Medical Sciences, 61-848 Poznan, Poland; 4 Integrated Science Association (ISA), Universal Scientific Education and Research Network (USERN), 60-806 Poznań, Poland

**Keywords:** blood–brain barrier, endothelium, fetal growth restriction, fetal hypoxia, neuronal damage, tight junctions, tight junction proteins

## Abstract

This study evaluated the damage to the endothelial tight junctions (TJs) in pregnancies complicated by fetal growth restriction (FGR) and investigated whether FGR is related to blood–brain barrier disintegration and, subsequently, to the appearance of proteins indicative of neuronal injury in maternal blood. The studied group included 90 pregnant women diagnosed with FGR. The control group consisted of 70 women with an uncomplicated pregnancy. The biochemical measurements included serum neuronal proteins (subunit of the N-methyl-D-aspartate receptor—NR1, nucleoside diphosphate kinase A—NME1, and S100 calcium-binding protein B—S100B), serum TJ proteins (occludin—OCLN, claudin-5—CLN5, zonula occludens—zo-1, and OCLN/zo-1 and CLN5/zo-1 ratios), and placental expression of TJ proteins (OCLN, claudin-4 CLN4, CLN5, zo-1). The significantly higher serum S100B and CLN5 levels and serum CLN5/zo-1 ratio were observed in FGR compared to healthy pregnancies. Moreover, FGR was characterized by increased placental CLN5 expression. Both serum NME1 levels and placental CLN4 expression in FGR pregnancies were significantly related to the incidence of neurological disorders in newborns. Mothers of FGR neonates who developed neurological complications and intraventricular hemorrhage (IVH) had statistically higher NME1 concentrations during pregnancy and significantly lower placental CLN4 expression than mothers of FGR neonates without neurological abnormalities. The serum NME1 levels and placental CLN4 expression were predictive markers of IVH in the FGR group. The blood–brain barrier is destabilized in pregnancies complicated by FGR. Neurological disorders, including IVH, are associated with higher serum concentrations of NME1 and the decreased placental expression of CLN4. The serum NME1 levels and placental CLN4 expression may serve as biomarkers, helpful in predicting IVH in FGR. It may allow for more precise monitoring and influence decision-making on the optimal delivery time to avoid developing neurological complications.

## 1. Introduction

Fetal growth restriction (FGR) is a pregnancy complication characterized by an inability of the fetus to reach its genetically predicted growth potential [1,2,3]. According to the criteria offered by Figueras and Gratacos, FGR is diagnosed when the estimated fetus weight (EFW) is lower than the 3rd percentile or, if the EFW is less than the 10th percentile, and a Doppler blood flow test indicates abnormalities associated with a poorer perinatal outcome [4]. FGR affects approximately 5 to 10% of pregnancies and is the second most common cause of perinatal mortality [5]. It has a multifactorial etiology, which is still not fully understood [6]. Its leading cause is a uteroplacental unit failure resulting in dysregulated blood flow across the placenta [7]. It is likely to result from an impaired trophoblast invasion of the uterine vasculature in the early stage of pregnancy, leading to abnormal conversion of the spiral arteries into low-resistance vessels that are limited in their delivery of nutrients and oxygen to the fetus [8,9,10]. A low birth weight is related to long-term consequences, evident from the in utero nervous system programming (Barker’s theory) [11,12,13,14]. FGR is associated not only with a higher risk of intrauterine fetal death, but also with poorer perinatal outcomes compared to those appropriate for gestational-age fetuses (AGA) [15,16,17].

The failure of invasive trophoblasts to sufficiently remodel the uterine arteries can lead to reduced blood flow, persistent placental hypoxia, and oxidative stress with consequences for fetal growth [18,19]. Initially, the fetus adapts to conditions of inadequate oxygen delivery, increasing cerebral, myocardial, and upper body blood flow while decreasing renal, gastrointestinal, and lower extremity perfusion. Circulatory centralization allows blood redistribution and preferential delivery of nutrients and oxygen to vital organs [20,21,22,23]. Eventually, as adaptive mechanisms are exhausted, the brain-sparing phenomenon does not provide sufficient protection against hypoxia for the developing fetal brain. It may lead to neurodevelopmental disorders on a heterogeneous spectrum [24,25,26].

Children with FGR show deficits in both brain function and structure [27]. FGR is associated with a reduction in total brain volume and gray matter, indicating its particular sensitivity to hypoxia and which is reflected in neurobehavioral impairments in children, including the ability to focus their attention [28,29,30]. In addition, postmortem studies of neonates with growth disorders have found a reduction in the total number of brain nerve cells [27,31]. Live-born FGR neonates show morphological differences in neurostructure, including abnormal corrugations of the cortex, and at 12 months of age, less structural complexity of the gray and white matter [27,32]. Additionally, prematurely born FGR neonates have lower global and local neural networks and reduced cortico-basal ganglia connectivity mainly in the prefrontal cortex and limbic system, compared to prematurely born neonates with AGA [27,33,34].

There are limited possibilities to study processes occurring in the developing fetal brain during pregnancy. Currently, the monitoring of a pregnancy complicated by FGR is based on repeated Doppler ultrasound and evaluation of cardiotocography records. There is a lack of sensitive and specific biomarkers of hypoxia in maternal blood that could be used in clinical practice for more precise monitoring of an FGR pregnancy to determine optimal delivery time and predict neonatal complications. Despite in vivo experimental studies and postmortem observations of fetuses with FGR, understanding the outcomes of placental insufficiency and in utero hypoxia on fetal neurodevelopmental processes is a challenge for modern perinatology. The sequence of events that occur in the brain during hypoxia is well understood, and these processes were observed in patients diagnosed with ischemic stroke [35,36].

The blood–brain barrier is a component of the neurovascular unit (NVU) that protects the nervous system from harmful agents and ensure the selective transport of substances from the blood to neurons. Under normal conditions, it is formed by tightly adherent endothelial cells, astrocytes, pericytes, the extracellular matrix, and the basal membrane [37,38,39]. The endothelial tight junctions (TJs) determine the paracellular permeability of molecules across the blood–brain barrier [40]. They consist of integral transmembrane proteins, i.e., occludin (OCLN), claudin (CLN), and junctional adhesion molecules (JAMs) [41], which are linked to the actin cytoskeleton by the zonula occludens proteins, zo-1, zo-2, and zo-3 [42]. Hypoxia-induced changes in blood–brain barrier permeability in pregnancies with FGR may result in the appearance of specific proteins in the blood that co-form the vascular or nonvascular (e.g., astroglial) part of the blood–brain barrier under normal conditions, the identification of which would allow monitoring of the pregnancy and a prognosis of neonatal complications. These markers could become a diagnostic tool to properly identify growth-restricted fetuses, among others, especially in those with mild abnormalities without severe placental insufficiency, which are more difficult both in terms of diagnosis and monitoring [43,44]. Given the hypothesis that abnormalities of TJs in FGR pregnancies are also reflected in processes occurring in the placenta, the searching for hypoxia indices in that maternal–fetal unit also seems justified.

The present study aimed to evaluate the usefulness of molecular indicators in identifying the damage to the endothelial TJs in pregnancies complicated by FGR and to investigate to what extend FGR is related to the release of blood–brain barrier proteins and molecules indicative of neuronal injury in maternal blood. The objectives were realized based on the evaluation of serum neuronal protein concentrations (subunit of the N-methyl-D-aspartate receptor—NR1, nucleoside diphosphate kinase A—NME1, and S100 calcium-binding protein B—S100B), serum levels (OCLN, claudin-5—CLN5, zonula occludens—zo-1, and OCLN/zo-1 and CLN5/zo-1 ratios), and the assessment of the placental expression of TJ proteins (OCLN, claudin-4—CLN4, CLN5, and zo-1). Moreover, the relationship between these biochemical parameters and neurological disorders in newborns was investigated. Furthermore, the usefulness of serum and placental proteins in predicting newborn neurological complications was evaluated.

## 2. Results

### 2.1. Group Characteristics

The basic characteristics of the studied and control groups, which did not differ in age, BMI at the first prenatal visit, and gestational age, are summarized in Table 1. The first, second, third, and fourth stage of FGR was diagnosed in 50.0, 12.2, 2.2, and 35.6% of patients, respectively. FGR newborns had significantly lower birth weights as compared to healthy neonates. They also revealed statistically lower values of all anthropometric measurements and lower body weight on the day of hospital discharge. FGR newborns had lower Apgar scores in the 1st and 5th minute and were hospitalized longer than healthy infants (Table 2).

### 2.2. Serum and Placental Markers

The significantly higher serum S100B and CLN5 levels and serum CLN5/zo-1 ratio were observed in FGR compared to the control pregnancy. Serum NR1, NME1, OCLN, and zo-1 levels and the serum OCLN/zo-1 index did not differ between groups (Table 3). The FGR group revealed significantly higher placental CLN5 expression. The placental expressions of OCLN, CLN4, and zo-1 were comparable in both groups (Table 4). A significant positive correlation between placental CLN5 expression and serum CLN5 levels was observed in the FGR group (Rs = 0.38, *p* = 0.0252).

### 2.3. Association of Serum and Placental Markers and Neurological Outcomes

**Figure 1:**
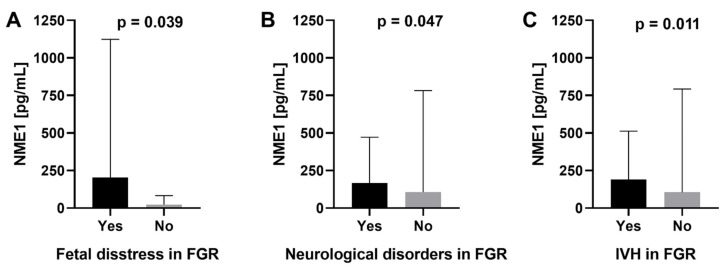
(**A**) Comparison of serum NME1 concentrations with reference to perinatal fetal distress in fetal growth restriction (FGR), (**B**) neurological disorders in FGR, and (**C**) intraventricular hemorrhage (IVH) in FGR newborns.

**Figure 2:**
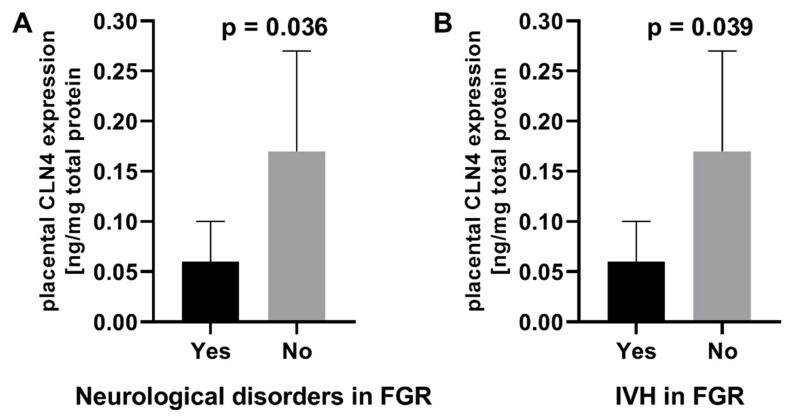
(**A**) Comparison of placental CLN4 expression with reference to neurological disorders in fetal growth restriction (FGR) and (**B**) intraventricular hemorrhage (IVH) in FGR newborns.

In FGR pregnancies, a significant relationship between serum NME1 levels and perinatal fetal distress was observed. Women from the FGR group, whose fetuses experienced life-threatening symptoms, had significantly higher serum NME1 concentrations compared to women with FGR without perilabour fetal distress (Figure 1A). Neurological disorders were observed in 8.9% of FGR newborns. They were not observed in children born from uncomplicated pregnancies. IVH and periventricular leukomalacia (PVL) were diagnosed in 7.8 and 2.2% of FGR neonates, respectively, with no cases in the control group. Mothers of FGR neonates who developed neurological complications had statistically higher NME1 concentrations during pregnancy (Figure 1B) and significantly lower placental CLN4 expression than mothers of FGR neonates without neurological abnormalities (Figure 2A). Moreover, mothers of FGR neonates diagnosed with IVH also showed significantly higher serum NME1 levels (Figure 1C) and lower placental CLN4 expression (Figure 2B) during pregnancy compared to mothers of FGR neonates without IVH.

The serum NME1 levels showed a prognostic value for IVH in the FGR group (cut-off value for NME1: 40.45 pg/mL; sensitivity: 0.71; specificity: 0.76; PPV: 0.21; NPV: 0.97; LR+: 2.93; LR-: 0.38; ACC: 0.753; AUC: 0.745; *p* = 0.0315). The placental CLN4 expression was useful in the prediction of newborn IVH (cut-off value for CLN4: 20.82 ng/mg total protein; sensitivity: 1.00; specificity: 0.83; AUC: 0.879; *p* < 0.0001).

## 3. Discussion

The present study is the first to provide data on serum concentrations of NR1, NME1, OCLN, CLN5, and zo-1 in human FGR pregnancies. Previously, only one study, which included a small group of patients, reported placental expressions of OCLN and zo-1 in pre-eclampsia with coexisting FGR [45]. Developing programs of neonatal neurocritical care (NNCP), which include disorders related not only to neonatal, but also peripartum and parturition periods, focus on diagnostic and monitoring procedures. The identification of fetal or neonatal neurological disorders requires neuroimaging, neurophysiological, and laboratory tools. “The First Thousand Days” approach was used by Michael Scher to define the Fetal/Neonatal Neurology Program. This program focuses on trimester-specific mechanisms that influence the maternal/placental/fetal (MPF) triad. Disturbing the MPF triad triggers maternal immune responses, which modify pre-neuronal and pre-glial cell populations and their interactions. As a result, embryonic/fetal central nervous system structures are lesioned, leading to a spectrum of neurological complications, e.g., encephalopathy of prematurity, cortical dysplasia, seizures, etc. [46]. The understanding of the mechanisms involved in the development of placental vasculature is required to identify manifestations of trimester-specific MPF associated with great obstetrical syndromes (GOSs): pre-eclampsia, FGR, prematurity, fetal demise, placental abruption, and morbidly adherent placenta. Moreover, the development of the neurovascular unit relies on the interaction between neurogenesis and angiogenesis. The proper course of pathways that leads to the final destinations requires interactions between vascular and neural components. A motor for an attractive or repulsive force forms as a result of intersensing between endothelial tip cell caps, which represent the vascular component, and the axon growth cone, which represents the neural component. Thus, already at the developmental stage of the blood–brain barrier, the involvement of angiogenesis in this process also indicates the significant role played later in blood–brain barrier function and integrity, where TJ proteins (i.e., OCLN, CLN, e-cadherin, zo, JAMs, catenins, cingulin, and actin) are crucial [47].

Our study adds to the general understanding of the potential association between indicators of neuronal lesions (NR1, NME1, and S100B), TJ/blood–brain barrier stability (OCLN, CLN5, and zo-1) and FGR, and its neurological outcomes in neonates with potential implications for clinical practice, early diagnosis, and management.

Animal studies do not provide a clear answer on the changes in TJ proteins under hypoxic conditions. Warrington et al. observed no altered expression of zo-1 and OCLN in either the anterior or posterior cerebrum in placental ischemic rats [48]. Kuvacheva et al. reported an increased number of cells with CLN5 expression and a decreased number of zo-1-positive cells after perinatal hypoxia in postpartum rats [49]. Ma et al. showed a downregulated expression of zo-1 and CLN5 in mouse-brain microvascular endothelial cells under hypoxia/aglycemia conditions [50]. Zehender et al. observed the destruction of zo-1 and CLN5 in hypoxia with subsequent reoxygenation [51], whereas Mark and Davis found changes in OCLN, zo-1, and zo-2 localization and an increased expression of these proteins during reoxygenation [52]. Similar to Mark and Davis, Yamagata et al. reported increased OCLN expression at the mRNA level [53]. Animal studies indicate the unquestionable effect of hypoxia on TJ protein distribution and point out that early life stress causes an imbalance between TJ protein expression, but the observed changes may be opposite in direction. This may require further studies on larger and more homogeneous groups to determine how reduced oxygen delivery affects endothelial TJ breakdown.

A similar increase in maternal serum and umbilical artery S100B levels in the FGR was independently observed in two previous studies and is in line with our observations [54,55]. Additionally, Gazzolo et al. determined the usefulness of S100B concentration in FGR pregnancy, with a cut-off value of 720 pg/mL, a sensitivity of 100%, and a specificity of 99.3% as a single marker to predict IVH in newborns. Moreover, S100B was suggested to be a potential marker for the early detection of IVH in infants with perinatal asphyxia before clinical examination and transtemporal ultrasound display pathological changes [56].

The incidence of neonatal IVH in our study was three-fold lower (7.8%) than reported by Gazzolo et al. [54]. Moreover, most newborns in our study presented with the first degree of IVH (5.6%), with an equal frequency of the second and third degrees (1.1%), and no occurrence of the fourth stage. These differences may be caused by the update of the guidelines for the management and recommended delivery time in FGR pregnancies. One should note that S100B levels in neonates with perinatal hypoxia reported by Gazzolo et al. are significantly higher than those observed in pregnancies with FGR. This suggests that the fetal blood–brain barrier breakdown, which results in the release of the S100B protein into the maternal bloodstream, is a dynamic process that progresses over time. Only its early detection, followed by decision-making about the optimal delivery time, may protect against neurological deficits in newborns. Contrary to this, Mazarico et al. and Boutsikou et al. did not report increased maternal S100B concentrations in FGR compared to physiological pregnancy [57,58]. This may have been because the researchers collected the maternal venous blood at the time of delivery. Moreover, they used the chemiluminescent immunoassay for the in vitro determination of serum S100B with an analytical range between 0.02 and 30 μg/l, whereas the limit of detection in our study was 5 pg/mL. Despite these discrepancies, S100B appears to be associated with FGR as its increased urine and serum levels were reported for FGR neonates, including those with neurological abnormalities detected one week after delivery [59,60]. All in all, S100B may be useful in the prediction of brain damage in neonates.

In addition, one study considered the possibility of a partial placental release of S100B in hypoxemic conditions because of its localization in intermediate villi and trophoblast cells. However, its expression does not change in pregnancies complicated by FGR and in physiological gestation [61]. Therefore, it is reasonable to assume that a significant increase in S100B in the fetus and/or in the placenta would lead to higher maternal blood levels, despite the increased total blood volume in pregnant women. It should be considered that S100B could be derived from maternal tissues [62], but all pregnant women included in the study were healthy and had no detectable neurological symptoms; thus, that possibility seems unlikely. We observed no relationship between the analyzed serum or placental proteins and perinatal outcomes in pregnant women. Thus, we can speculate that the changes in the placental expression of TJ proteins and their associations with neurological complications in the newborn reflect processes associated with fetal blood–brain barrier destabilization in pregnancies complicated by FGR.

The present study provides novel information on serum NME1 as a valuable marker in IVH prediction among FGR newborns. Furthermore, significantly higher NME1 concentrations were observed in FGR pregnancies when perinatal fetal distress occurred, indirectly suggesting the association of this protein with neuronal damage. Although serum NR1 concentrations showed no difference between FGR and physiological pregnancies, it oscillated close to the significance limit, with a predominance of higher values in pregnancies complicated by FGR. NR1 is one of the subunits of N-methyl-D-aspartate receptors (NMDAR), which is reported to decrease in response to prenatal hypoxia (to which the fetal brain is particularly vulnerable). That results from the high expression and activity of these receptors in fetuses, which are specific roles of NMDAR neurotransmission in the maturation and plasticity of developing neurons, and which change the NMDAR configuration or their affinity to neurotransmitters in response to noxious stimuli [63,64]. Therefore, we hypothesized that a decrease in the expression of these receptors in response to intrauterine hypoxia could occur with the persistence of adverse conditions during fetal development in utero. At the time of blood collection from pregnant women, the disorder may have been moderately severe, as indicated by the predominance of the first stage of FGR diagnoses. Therefore, the disintegration of the blood–brain barrier could already be identified (as evidenced by significantly higher serum CLN5 and S100B concentrations and the serum CLN5/zo-1 index) with a relative increase in NR1 in maternal blood. However, over time, as the Doppler blood flow worsens, a significant change in levels of NR1, as well as NR2 and NR3, could occur due to the degradation of NMDAR. These hypotheses were put forward by Schober et al. and Phillips et al., who observed the reduction in the NR1 subunit and NR2A to NR2B ratio, dendrite shortening, and the reduction in the density of immunostaining of NR1 due to hypoxia in rats with induced growth restriction because of placental insufficiency [65,66]. Understanding the role of the NMDAR and the balance between their different subunits in FGR pregnancy requires further research, perhaps including the role of their agonists and antagonists.

Our study did not observe any difference in OCLN expression, which is in line with some previous research on pre-eclampsia and laboratory-induced hypoxia [67,68]. Contrary to this, Lim et al. and Wang et al. reported a decrease in OCLN expression at the protein level, although there were no changes in mRNA expression in patients diagnosed with pre-eclampsia compared to physiological pregnancy [45,69]. Moreover, pre-eclampsia coexisted with a disorganized pattern of TJ proteins [69]. Similar to our study, Lim et al. and Liévano et al. did not observe changes in zo-1 expression [45,67], whereas Zhang et al. noticed a decrease in zo-1 and CLN4 expression, as well as an increase in CLN8 expression [68]. Contrary to the increase in CLN5 expression observed in the present study, Liévano et al. found a decrease in CLN5, CLN1, and CLN3 expression [67]. This may suggest the existence of further pathways which control the placental claudin expression and distinct mechanisms of “up-” and “down-” regulation of TJ proteins in response to the conditions in utero. Zhang et al. concluded that placental TJ dysfunction induced by reduced oxygen concentrations might be important in the pathogenesis of pre-eclampsia [68].

Similarly, in our study, along with increased placental CLN5 expression, significantly higher serum CLN5 levels were observed in the FGR group. This suggests that the disorganization of placental TJ proteins, contributing to reduced endothelial cell tightness, may influence further blood flow deterioration, leading to placental insufficiency and affecting fetal blood–brain barrier stability with the subsequent release of CLN5 into the maternal blood circulation. It remains significant that CLN5 is localized in the blood–brain barrier on the outer part of the endothelial cell membrane with the internal organization of OCLN and zo-1. Thus, the observed relationship may be derived from partial destabilization of TJ proteins in the developing fetal brain, resulting in a higher release of CLN5 into the maternal blood compared to other TJ proteins or indicating the involvement of additional molecules supporting these connections. The lower placental expression of CLN4 was found in FGR pregnancies with neurological disorders of newborns, including IVH. Since the multinucleated syncytiotrophoblast layer shows the presence of CLN4 in the basolateral part of the cell membrane [67], it can be speculated that the disorganization of the placental TJ proteins, leading to lower CLN4 expression, may be related to newborn neurological complications, including IVH. Moreover, it should be pointed out that the finding of significantly higher serum NME1 concentrations in pregnant women with FGR was also associated with neurological abnormalities and IVH. Because Lööv et al. reported the appearance of NME1 only in cultures of damaged neurons and, therefore, suggested the neuroprotective or regenerative function of extracellular NME1 as well as the lack of its secretion in normal conditions [70], the link between the change in NME1 levels in pregnant patients with FGR and the secondarily found reduced placental CLN4 expression seems reasonable. A limitation of the study was the lack of sequential analysis in the newborns’ urine, which could show the changes in the studied parameters occurring during the delivery period and postnatal life. Perhaps the studied parameters could be used in the prognosis of the later abnormalities found through transcranial ultrasound. Moreover, the studied groups, although homogeneous, were relatively small. In addition, the long-term neurological consequences were not evaluated. The final limitation of our study is the inability to exclude the subtle lesions in the placenta, such as those from the maternal or fetal inflammatory response to an ascending intrauterine infection, the diagnosis of which may not be available through conventional histopathological detection [71].

## 4. Materials and Methods

### 4.1. The Studied and Control Groups

The study was conducted in collaboration between the Department of Perinatology and Gynecology and the Department of Neurochemistry and Neuropathology of the Poznan University of Medical Sciences between 2015 and 2019.

The studied group included 90 pregnant women between 24 and 41 weeks of gestation and who were diagnosed with FGR according to the Figueras and Gratacós criteria [4]. The control group comprised 70 women with an uncomplicated pregnancy between 29 and 41 gestational weeks. Before inclusion in the study, a detailed medical interview was conducted regarding obstetric history, course of current pregnancy, chronic diseases, and medications. Then, each pregnant woman underwent an obstetric examination and Doppler ultrasound velocimetry (Voluson E10 BT18, GE Healthcare, Chicago, IL, USA). All newborns underwent a routine neonatal examination after delivery, but in the cases of severe asphyxia, it was performed after the stabilization of the vital functions. The neurological examination included an evaluation of a neonate’s level of alertness, cranial nerve function, sensory and motor system function, and the presence of primitive reflexes. In the case of an infant born with less than 32 weeks of gestation, the first transcranial ultrasound examination was not performed until the 3rd day of life, and the second examination between the 5th and 7th days after delivery. If IVH was diagnosed using Papile’s classification [72,73], the frequency of subsequent examinations depended on identifying primary changes and the baby’s clinical condition. If the ultrasound image was unclear, a magnetic resonance (MR) was performed. MR was performed at the postconceptional age between 38 and 42 weeks if no changes were observed. In the case of a newborn between 32 and 35 weeks of gestation, an ultrasound was performed at the same time intervals as mentioned above, and if an abnormal or unclear image was found, MR was performed. If the neonate, born after 35 weeks of gestation with an Apgar score between 0 and 3 or an umbilical cord blood pH below 7.0, received therapeutic hypothermia, a Doppler ultrasound followed by MR was performed between the 7th and 10th day after delivery. If therapeutic hypothermia was not applied, ultrasound examinations were repeated until the 3rd day of life and between the 5th and 7th day after delivery. MR was performed when neurological abnormalities were observed. PVL was diagnosed using MR [74,75]. In newborns from physiological pregnancies born at term, because of the absence of risk factors for central nervous system damage and no neurological abnormalities at birth, diagnostic imaging was not necessary.

The exclusion criteria were as follows: maternal malnutrition, nicotinism, alcohol consumption, drug abuse, taking medications such as warfarin, antiepileptic drugs, anticancer drugs, folic acid antagonists (trimethoprim-sulfamethoxazole, phenobarbital), cyanotic congenital heart defects, heart failure NYHA III/IV, uncontrolled asthma, chronic obstructive pulmonary disease, cystic fibrosis, pregestational and gestational diabetes mellitus, chronic renal failure, nephrotic syndrome, renal transplantation, continuous hemodialysis, systemic lupus erythematosus, antiphospholipid syndrome, Crohn’s disease, ulcerative colitis, severe anemia, sickle cell anemia, beta-thalassemia, hemoglobin H disease, and uterine malformations. Fetal exclusion factors were chromosomal aberrations, autosome abnormalities, uniparental disomies, microdeletion syndromes, congenital malformations, and confirmed infection with cytomegalovirus, rubella virus, herpes simplex virus, varicella-zoster virus, human immunodeficiency virus, *Toxoplasma gondii*, *Treponema pallidum*, *Chlamydia* sp., *Mycoplasma* sp., *Listeria monocytogenes*, or *Mycobacterium tuberculosis*. The women with placenta previa, placenta accreta, placental infarcts, placental villous thrombosis, circumvallate placenta, hemangiomas, or other placental tumors were also excluded from the study.

### 4.2. Collection of Blood and Placental Samples

Three tubes per clot, each with 7.5 mL of whole blood, were collected from each patient and centrifuged for 10 min at 2750× *g*. The serum was transferred into Eppendorf tubes and frozen at −80 °C. The external section of the placental plate, 25 cm^2^ in size, was taken from the opposite side of the umbilical cord. If the umbilical cord was centrally located, the external sample of the placental plate was cut, regardless of the location. Fresh tissue was frozen at −80 °C.

### 4.3. Laboratory Serum Assays

The commercial ELISA assays were applied to measure the levels of NR1 (Human Glutamate [NMDA] receptor subunit zeta-1, GRIN1 ELISA Kit, MyBioSource, San Diego, CA, USA), NME1 (Human Nucleoside diphosphate kinase A, NME1 ELISA Kit, MyBioSource, San Diego, CA, USA) and S100B (S100B human ELISA kit, DRG MedTek, Warsaw, Poland). Because of the lack of commercial diagnostic tests for OCLN, zo-1, and CLN5, these concentrations were assessed using an in-house ELISA method. The Nunc MaxiSorpTM plates (Thermo Fisher, Waltham, MA, USA) were used for all measurements. The rabbit anti-human and mouse anti-human antibodies were used as capture and detection antibodies for the determination of serum OCLN levels (Occludin Polyclonal Antibody, Zymed, South San Francisco, CA, USA, RRID AB_2533977; Occludin Monoclonal Antibody (OC-3F10), Invitrogen, Waltham, MA, USA, RRID AB_2533101) and zo-1 levels (ZO-1 Polyclonal Antibody, Zymed, South San Francisco, CA, USA, RRID AB_2533938; ZO-1 Monoclonal Antibody (ZO1-1A12), Invitrogen, Waltham, MA, USA, AB_2533147), respectively. For CLN5, mouse anti-human antibodies (Claudin 5 Monoclonal Antibody (4C3C2), Zymed, South San Francisco, CA, USA, RRID AB_2533200) were used as capture antibodies, whereas rabbit anti-human antibodies (Claudin 5 Polyclonal Antibody, Abcam, Cambridge, UK, RRID AB_2533157) were used for detection. Goat anti-mouse IgG (Goat anti-Mouse IgG (H+L) Cross-Adsorbed Secondary Antibody, HRP, Invitrogen, Waltham, MA, USA, RRID AB_2536527) served as the secondary antibodies for OCLN and zo-1, whereas goat anti-rabbit IgG (H+L, HRP, Invitrogen, Waltham, MA, USA) was used for CLN5. The Substrate Reagent Pack (Substrate Reagent Pack, R&D Systems™, Minneapolis, MN, USA) was used in that reaction. Recombinant human OCLN (Recombinant Human Occludin GST (N-Term) Protein, Novus Biologicals, Littleton, CO, USA) and recombinant human CLN5 (Recombinant Human Claudin-5 GST (N-Term) Protein, Novus Biologicals, Littleton, CO, USA) served as standards. The concentrations of all tested factors, except zo-1, were expressed in pg/mL. Due to the lack of an acceptable standard for zo-1, relative units (RUs) were calculated from the optical density (OD), measured at 450 nm (OD450) as the quotient: OD of 10 samples per OD of the cut-off. The OD was statistically determined from zo-1 serum measurements of 48 healthy patients, whereas the 95th percentile was defined as the cut-off value. The serum zo-1 concentration was expressed in RU/mL. All ELISA steps were conducted using an automated RT-3100 microplate washer (Rayto Life and Analytical Sciences Co., Ltd., Shenzhen, China), and the final reading was performed using an EL×800 microplate reader (BioTek, Winooski, VT, USA). Since blood–brain barrier disintegration is associated with the release of TJ proteins, externally expressed CLN5 and internally expressed OCLN and zo-1, the ratios of OCLN/zo-1 and CLN5/zo-1 were calculated to estimate the degree of blood–brain barrier breakdown.

### 4.4. Laboratory Placental Tests

Placental tissues were homogenized in a buffer (1 L) consisting of 150 mM NaCl, 5 mM ethylenediaminetetraacetic acid (EDTA), and 50 mM Tris buffer solution (all Sigma-Aldrich, Saint Louis, MO, USA). The mixture of protease inhibitors and Triton X-100 (both Sigma-Aldrich, Saint Louis, MO, USA) was added to the buffer, giving a solution with a final concentration of 1%. The protease inhibitors included: fluorinated 4-(2-aminoethyl)benzenesulfonyl hydrochloride (AEBSF), aprotinin, bestatin hydrochloride, trans-epoxysuccinyl-L-leucylamido(4-guanidino)butane (E-64), EDTA, and leupeptin hemisulfate. The tissue homogenates were centrifuged for 15 min in Eppendorf tubes at 10,000 rpm. The obtained filtrate was used for TJ protein expression analyses. All ELISA steps were performed using an automated microplate washer (RT-3100 Microplate Washer, Rayto Life and Analytical Sciences Co., Ltd., Shenzhen, China), and the records were read out using an EL×800 microplate reader (BioTek, Winooski, VT, USA). The placental expression of TJ proteins was estimated by using the Lowry method and described as ng/mg of total protein [76]. The dependence of the absorbance of the comparison solutions on protein concentration was plotted, and linear regression was used to prepare a standard curve. The protein concentration was determined from the standard curve and the absorbance of the test solution [77]. The placental expression of OCLN, zo-1, and CLN5 was analyzed using an in-house ELISA (described in Section 2.3. Laboratory Serum Assays). The CLN4 expression was evaluated by using a commercial ELISA kit (ELISA Kit for Claudin 4 (CLDN4), USCN Life Science, Wuhan, China).

### 4.5. Statistical Analysis

The statistical analysis was performed with Statistica StatSoft 13.1 (StatSoft, Krakóv Poland) and PQStat 1.8.0 (PQStat, Warsaw, Poland). The normality of the data distribution was checked using Kolmogorov–Smirnov, Lilliefors, and Shapiro–Wilk tests. If the assumption of the Gaussian distribution was met, Student’s *t*-test was used for calculations; otherwise, the nonparametric U-Mann–Whitney test was performed. The chi-square test and Fisher’s exact test were used to analyze the data expressed on a nominal scale. The correlations were assessed with Spearman’s rank correlation coefficient (Rs). The usefulness of serum and placental measurements in the prognosis of neurological disorders was evaluated with a receiver operating curve (ROC). The prediction analysis included area under the curve (AUC), sensitivity, specificity, positive predictive value (PPV), negative predictive value (NPV), reliability quotient of the positive result (LR+), reliability quotient of the negative result (LR-), and accuracy (ACC), calculated using the DeLong’s nonparametric method and the Clopper–Pearson method for a single proportion and cut-off value. A *p*-value < 0.05 was considered statistically significant.

## 5. Conclusions

The present study indicates that the blood–brain barrier is destabilized in pregnancies complicated by FGR and marked by increased serum levels of CLN5 and S100B and the CLN5/zo-1 ratio in maternal blood. The neurological complications in FGR, including IVH, are associated with the increased release of NME1 into the maternal blood and decreased placental CLN4 expression. The serum NME1 and placental CLN4 expression may be predictive markers of IVH in FGR. They may allow for more precise monitoring and facilitate decision-making about the optimal delivery date to avoid fetuses developing neurological complications. Further research is needed to consider the time of FGR diagnosis with groups divided into early- and late-onset FGR or regarding fetal circulatory centralization (brain sparing). Moreover, the follow-up of FGR newborns could provide valuable data on long-term neurological deficits.

## Figures and Tables

**Table 1:** The characteristics of the group with fetal growth restriction (FGR) and physiological pregnancy.

Characteristics	FGR (*n* = 90)	Physiological Pregnancy (*n* = 70)	*p*-Value
Age (years, mean ± SD)	29 ± 5	31 ± 5	0.0662
BMI at the first prenatal visit (kg/m^2^) median, min–max)	22.2 (15.2–42.0)	21.9 (17.4–30.5)	0.8443
Gestational age at study eligibility (weeks, median, min–max)	35 (24–41)	36 (29–41)	0.0677
Estimated fetal weight (g, median, min–max)	1932 (439–2920)	3255 (756–4259)	**<0.0001**
Percentile of estimated fetal weight (median, min–max)	1.0 (0.1–9.0)	41.0 (4.0–98.0)	**<0.0001**
Mode of delivery (%)			
Spontaneous	20.5	41.8	**0.0039**
Cesarean section	75.0	41.8	**<0.0001**
Vacuum extractor	4.5	16.4	**0.0132**
Forceps	0.0	0.0	1.0000

bold values means statistical significance at the *p* < 0.05 level.

**Table 2:** The perinatal outcomes of newborns in pregnancies complicated by fetal growth restriction (FGR) and physiological pregnancy. The statistically significant differences between groups are highlighted in bold.

Parameters	FGR (*n* = 90)	Physiological Pregnancy (*n* = 70)	*p*-Value
Delivery week (median, min–max)	37 (26–41)	39 (37–42)	**<0.0001**
Premature delivery (%)	42.2	0.0	**<0.0001**
Fetal distress (%)	50.0	20.0	**0.0001**
Birth weight (g) (median, min–max) <2500 overall (%) 1500–2500 (%) 1000–1500 (%) <1000 (%)	2260 (420–3080) 70.5 46.6 8.0 15.9	3450 (2500–4600) 0.0 0.0 0.0 0.0	**<0.0001**
Anthropometric measurements (cm) (median, min–max)			
Head circumference	31.5 (22.0–35.0)	34.0 (31.0–38.0)	**<0.0001**
Thoracic circumference	29.0 (17.5–33.0)	34.0 (27.0–37.5)	**<0.0001**
Body length	49.0 (29.5–55.0)	55.0 (45.0–61.0)	**<0.0001**
Apgar score (points) (median, min–max)			
1st minute	10 (0–10)	10 (4–10)	**0.0017**
3rd minute	8 (2–10)	9 (6–9)	0.6209
5th minute	10 (4–10)	10 (9–10)	**0.0001**
pH (median, min–max) Venous Arterial	7.33 (7.01–7.46) 7.27 (6.95–7.45)	7.33 (7.15–7.48) 7.27 (7.06–7.40)	0.5835 0.9758
Base excess (mEq/L) (median, min–max) Venous Arterial	−2.6 (−11.3–3.2) −2.3 (−13.4–3.4)	−2.7 (−10.3–2.7) −2.6 (−11.8–3.4)	0.2271 0.1345
Metabolic acidosis (%)			
Arterial pH < 7,30 overall	56.7	42.9	0.1108
7.20–7.29	42.2	30.0	0.1376
7.10–7.19	12.2	10.0	0.8023
7.00–7.09	0.0	2.9	0.1899
<7.00	2.2	0.0	0.5047
Length of hospitalization (days) (median, min–max)	6 (3–84)	4 (2–22)	**<0.0001**
>5 days (%)	50.6	14.5	**<0.0001**
Neurological disorders overall (%)	8.9	0.0	**0.0098**
Intraventricular hemorrhage (%)	7.8	0.0	**0.0186**
First degree	5.6	0.0	0.0683
Second degree	1.1	0.0	1.0000
Third degree	1.1	0.0	1.0000
Fourth degree	0.0	0.0	-
Periventricular leucomalacia (%)	2.2	0.0	0.5044

bold values means statistical significance at the *p* < 0.05 level.

**Table 3:** Serum concentrations of biochemical parameters in pregnancies complicated by fetal growth restriction (FGR) and physiological pregnancy. The statistically significant differences between groups are highlighted in bold.

Serum Concentrations	FGR (*n* = 90)	Physiological Pregnancy (*n* = 70)	*p*-Value
NR1 (pg/mL) mean ± SD (min–max)	1295.2 ± 2852.6 (0.0–16,442.1)	657.2 ± 1531.2 (0.0–9030.8)	0.0799
NME1 (pg/mL) mean ± SD (min–max)	112.4 ± 650.7 (0.0–6085.6)	77.1 ± 317.3 (0.0–2557.8)	0.9246
S100B (pg/mL) mean ± SD (min–max)	29.6 ± 40.7 (0.0–165.9)	14.9 ± 25.1 (0.0–72.8)	**0.0290**
OCLN (pg/mL) mean ± SD (min–max)	32.8 ± 116.3 (0.0–676.0)	8.4 ± 41.9 (0.0–284.0)	0.0835
CLN5 (pg/mL) mean ± SD (min–max)	74.2 ± 167.6 (0.0–828.0)	30.7 ± 150.6 (0.0–1115.0)	**0.0039**
zo-1 (RU/mL) mean ± SD (min–max)	2.6 ± 4.6 (0.0–24.9)	1.8 ± 4.2 (0.0–29.3)	0.3122
OCLN/zo-1 (pg/RU) mean ± SD (min–max)	11.4 ± 50.5 (0.0–320.9)	1.7 ± 8.9 (0.0–48.1)	0.0612
CLN5/zo-1 (pg/RU) mean ± SD (min–max)	122.8 ± 6378.0 (0.0–4127.0)	4.7 ± 12.2 (0.0–40.8)	**0.0181**

bold values means statistical significance at the *p* < 0.05 level.

**Table 4:** Placental expression of tight junction proteins in pregnancies complicated by fetal growth restriction (FGR) and physiological pregnancy.

Placental Expression	FGR (*n* = 90)	Physiological Pregnancy (*n* = 70)	*p*-Value
OCLN (ng/mg total protein) mean ± SD (min–max)	0.18 ± 0.16 (0.00–0.53)	0.12 ± 0.13 (0.00–0.43)	0.0791
CLN5 (ng/mg total protein) mean ± SD (min–max)	0.02 ± 0.02 (0.00–0.11)	0.01 ± 0.02 (0.00–0.09)	**0.0119**
CLN4 (ng/mg total protein) mean ± SD (min–max)	0.16 ± 0.10 (0.00–0.42)	0.13 ± 0.10 (0.02–0.43)	0.1806
zo-1 (RU/mL) mean ± SD (min–max)	0.24 ± 0.16 (0.02–0.66)	0.20 ± 0.16 (0.02–0.54)	0.2615

bold values means statistical significance at the *p* < 0.05 level.

## Data Availability

The data presented in the study are available from the corresponding author upon reasonable request.
